# Abnormal Blink Reflex and Intermuscular Coherence in Writer's Cramp

**DOI:** 10.3389/fneur.2018.00517

**Published:** 2018-07-02

**Authors:** Supriyo Choudhury, Ravi Singh, Payel Chatterjee, Santosh Trivedi, Shantanu Shubham, Mark R. Baker, Hrishikesh Kumar, Stuart N. Baker

**Affiliations:** ^1^Department of Neurology, Ram Gopal Chamaria Research Center, Institute of Neurosciences, Kolkata, India; ^2^Department of Neurology, Royal Victoria Infirmary, Newcastle upon Tyne, United Kingdom; ^3^Department of Clinical Neurophysiology, Royal Victoria Infirmary, Newcastle upon Tyne, United Kingdom; ^4^Institute of Neurosciences, The Medical School, Newcastle University, Newcastle upon Tyne, United Kingdom

**Keywords:** pre-pulse inhibition, oscillations, theta band, writer's cramp, focal dystonia

## Abstract

**Background:** Writer's cramp (WC) is a task-specific focal hand dystonia presenting with pain, stiffness and/or tremor while writing. We explored the involvement of cortical and brainstem circuits by measuring intermuscular coherence (IMC) and pre-pulse inhibition (PPI) of the blink reflex.

**Methods:** IMC was measured in 10 healthy controls and 20 WC patients (10 with associated tremor) while they performed a precision grip task at different force levels. Blink responses were evaluated in 9 healthy controls and 10 WC patients by stimulating the right supraorbital nerve and recording surface EMG from the orbicularis oculi muscles bilaterally. PPI involved conditioning this stimulation with a prior shock to the right median nerve (100 ms interval), and measuring the reduction in the R2 component of the blink reflex.

**Results:** Significant IMC at 3–7 Hz was present in WC patients, but not in healthy controls. Compared to healthy controls, in WC patients the R2 component of the blink reflex showed significantly less PPI. IMC at 3–7 Hz could reliably discriminate WC patients from healthy controls.

**Conclusion:** Cortical or sub-cortical circuits generating theta (3–7 Hz) oscillations might play an important role in the pathogenesis of WC. Moreover, the lack of PPI implicates abnormalities in brainstem inhibition in the emergence of WC. IMC may merit further development as an electrodiagnostic test for focal dystonia.

## Introduction

Writing is a highly developed motor task requiring fractionated contraction of hand and forearm muscles. In some, this fine balance of activity is lost, writing becomes increasingly difficult and a disorder popularly known as writer's cramp (WC) emerges. These patients usually present with difficulty in writing, abnormal posturing, painful spasms and muscle cramps or tremor affecting the forearm or hand whilst writing but not during other activities involving the same muscle groups. These signs are associated with co-contraction of antagonistic muscles (1). WC is more formally classified as a task specific focal hand dystonia (2, 3), of which there are other examples reported in typists, musicians and golfers (4). In some patients with WC, dystonic posturing is almost imperceptible and there is focal tremor more prominent whilst writing, similar to dystonic tremor (5).

Focal dystonia is thought to emerge because of the loss of central inhibition at various anatomical levels including cerebral cortex, brainstem and spinal cord (6). In WC, given the importance of the corticospinal tract in the control of skilled finger and hand movements such as writing (7), loss of inhibition within sensorimotor cortex is thought to be the almost exclusive cause of the dystonia (6, 8). This supposition is supported to some extent by the lack of strong evidence of brainstem involvement using techniques that are able to probe brainstem physiology non-invasively. One such technique is pre-pulse inhibition (PPI) of the blink reflex. In healthy people, the blink reflex is normally inhibited if conditioned by a preceding peripheral nerve stimulus, a phenomenon referred to as PPI. This is thought to be mediated via an inhibitory brainstem reticular pathway involving the pedunculo-pontine nucleus (PPN), and is typically diminished in disorders affecting the PPN or adjoining structures (9, 10). Whilst PPI of the blink reflexes is reportedly abnormal in craniofacial dystonia (11), cervical dystonia and blepharospasm (12, 13), in a previous study of 3 WC patients PPI was normal (8).

Intermuscular (EMG-EMG) and corticomuscular (EEG-EMG) coherence (IMC, CMC) have both been used extensively to describe changes in brain connectivity in various movement disorders (14). Moreover, there is growing evidence that coherence at specific frequencies is mediated via specific pathways. For example beta-band (15–30 Hz) coherence is dependent on an intact corticospinal tract (15). IMC has also been investigated in patients with dystonia, and increased theta-band (3–7 Hz) IMC was observed in some DYT1 positive patients with upper limb dystonia (16). One study (17) used MEG in patients with WC and the technique of dynamic imaging of coherent sources (DICS). This identified significant coherence in the 3–7 Hz (theta) range between upper limb EMG and ipsilateral cerebellum, contralateral thalamus and bilateral sensorimotor cortex. This provided further support to the notion that WC is driven by dysfunctional cortical networks involved in hand control.

Here we present results of experiments in patients with WC from an Indian tertiary referral center. This has provided an opportunity to re-assess the circuits contributing to the pathophysiology of WC by investigating PPI and intermuscular coherence (IMC) in a large cohort of patients.

## Methods

Experiments were conducted in 38 participants (22 WC patients, 16 healthy control) at a tertiary care neurology center in Eastern India and approved by the Institutional Research Ethics Committee. Informed consent was obtained from each participant prior to the study in accordance with the Declaration of Helsinki. Twenty two male patients (mean age 55 years, SD 10.5 years) had Writer's Cramp (WC). Twenty WC patients participated in IMC experiments and 10 participated in blink experiments (8 WC patients participated in both experiments). WC patients were diagnosed according to the Movement Disorders Society (MDS) consensus update on the classification of dystonia (18). Ten WC patients had clinically observable tremor while writing, whereas the rest of the patients presented without visible tremor, only with posturing. Secondary causes of tremor or dystonia were excluded by clinical examination and standard clinical investigations. For example, brain imaging was used to look for structural/vascular causes and electroneurography for neuropathic tremor. Patients who had been treated with botulinum toxin were also excluded from the study. Patients were either treatment naïve or tested after a 10-day washout of neuro-psychiatric medications (>5 half-lives for concomitant medication). The medications were baclofen, trihexyphenidyl, clonazepam, escitalopram either alone or in combination. We also recruited 16 healthy participants (10 participated in the IMC study and 9 in the blink reflex study) as controls [age range 25–67 years; mean (SD) age for blink experiment: 50 (9.91) years, for coherence experiment: 36 (13.07) years]. All the WC patients had dystonic posturing of the fingers, wrist or elbow while writing. The severity of WC was assessed through the Writer's Cramp Rating Scale (WCRS, 19). The selection of participants for coherence and blink experiments were as per the convenience and the consent of the subjects.

### Recordings

For coherence experiments electromyogram (EMG) signals were recorded from superficial wrist and finger flexors, extensors and first dorsal interosseous muscles (FDI), using adhesive surface electrodes (H59P, Kendall) placed over the muscle belly with an inter-electrode spacing of 2 cm for all the muscles. EMG recordings in patients and controls were made from the right side, which in patients was the affected side.

In experiments investigating PPI of the blink reflex by median nerve stimulation, surface EMG was recorded from orbicularis oculi (OO) bilaterally, using the same adhesive electrodes placed on the lower eyelid with the reference on the lateral canthus (bipolar montage), and a ground on the lateral forehead.

EMG electrodes were connected to a custom-built isolated amplifier (gain x1,000, bandpass 30Hz−2 kHz). Amplified potentials together with signals from the force transducer and event markers were digitized via a power1401 interface (Cambridge Electronic Design, Cambridge, UK) at a sampling frequency of 5,000 Hz and saved to hard disk for off-line analysis.

### Pre-pulse inhibition (PPI)

PPI of the blink reflex was investigated in 10 patients with WC and 9 age matched healthy participants.

The right (all patients were right handed) supraorbital nerve was stimulated (0.2 ms pulse width) via two adhesive surface electrodes, placed on the forehead over the supraorbital notch (slightly medial to the upper border of the superior orbital margin) with an inter-electrode distance of 1 cm (anode above cathode), connected to a constant current stimulator (DS7A; Digitimer). The minimal perceptible sensory threshold was recorded for every participant. The appearance of a deflection of the OO EMG 40–60 ms after supraorbital nerve stimulation defined threshold for the blink reflex. Stimulation intensity for the supra-orbital nerve was adjusted to a level above this threshold to elicit a clear blink reflex.

The median nerve was stimulated (1.8–5.4 mA; 0.2 ms pulse width) using adhesive electrodes (see above) placed on the volar aspect of the wrist of the right hand and connected to a DS7A (Digitimer) constant current stimulator. The current strength for median nerve stimulation was set at 3x minimum perceptible sensory threshold. Twenty stimuli to the supraorbital nerve were delivered at a fixed inter-stimulus interval of 20 s. Half of the supraorbital nerve stimuli (chosen in pseudo-random order) were conditioned by a preceding median nerve shock, delivered 100 ms before supraorbital nerve stimulation.

### Coherence task

Participants were seated in a comfortable chair with the dominant forearm resting on a writing board, holding a transducer between index finger and thumb which measured pinch grip force (Biometrics Ltd, Newport, UK). The force exerted at maximum voluntary contraction (MVC) was measured for every participant at the start of the session. Subjects then performed a task which required them to squeeze, hold and release the force transducer, producing isometric contractions. The required force was indicated to the subject by a target on a computer screen, and was set to 10, 20, 40, 60, or 80% of MVC. Each contraction was initiated by the target moving to the appropriate force level, and a brief beep. The subject then had to squeeze to move a cursor (indicating measured force) rapidly into the target for a 3 s hold period. At the end of this time, the target returned to the bottom of the screen (corresponding to zero force), and another beep cued the subject to relax. Trials were separated by 2 s. Force levels were presented in random order, with 30 trials per level giving a total of 150 trials. After 50 and 100 trials, a 2 min rest period was provided. Data acquisition and the control of audio and visual feedback were performed using custom scripts written in Spike2 software (Cambridge Electronic Design, UK). All WC patients (and 10 controls) completed the IMC study. We have previously shown that there are no age and gender dependent changes in IMC amplitude, so that we did not consider it necessary to match age and gender for the control group for this experiment (19).

### Analysis

Blink responses were analyzed by forming averages of full-wave rectified OO EMG triggered by the supraorbital nerve stimulus. A bilateral R2 response (onset latency around 50 ms) was consistently present in all participants. Reflex amplitude was measured by calculating the area under the curve above baseline between the onset and offset times; these times were placed by the user at the points where the response rose above, or fell below, three standard deviations above the mean rectified background EMG. The ratio of conditioned to unconditioned reflex amplitude served as an index of paired pulse inhibition.

Coherence analysis followed the procedures used in our prior publications (20, 21). Analysis focused on a region 0.6–3.06 s after the cue indicating trial onset; this region was chosen as containing relatively stationary contractions, during which we know beta frequency (15–25 Hz) oscillations are greatest. EMG power spectra and IMC were calculated using three contiguous 0.82 s-long (4,096 sample points) sections of data from each trial, yielding a frequency resolution of 1.22 Hz (22). EMG signals were full-wave rectified before Fourier transforms were calculated. Significance levels were calculated using the method described in Rosenberg et al. (23). IMC was calculated between the FDI muscle and both flexor and extensor muscles. By confining our analysis to these muscle pairs, which are physically well separated, we avoided any contribution of electrical cross-talk to the measured IMC (24).

IMC values were reported in two ways. Firstly, we averaged spectra for a given muscle pair across all patients within a group, providing a visual representation of the dependence of coherence on frequency. Significance limits for these averaged spectra were computed as we have described previously (25, 26). The coherence in a frequency band between two groups was compared using unpaired Student *t*-tests (parametric data). Secondly, we calculated the mean coherence across the theta-frequency (3–7 Hz) and beta-frequency (15–30 Hz) bands for a given muscle pair in single subjects. This allowed us to estimate the distribution of mean coherence in given frequency bands across the population. Cumulative distribution plots of IMC across subjects were plotted, and from these receiver operating characteristic (ROC) curves constructed, which indicate whether a measure might have utility as a diagnostic tool. ROC curves were summarized by computing the area under the curve (AUC), which does not require any assumptions regarding the underlying distribution. An AUC of 0.5 would indicate an identical distribution of the measure of interest in patients and controls; an AUC of 1.0 would occur if the distributions were entirely non-overlapping. We determined whether the AUC was significantly larger than 0.5 using a Monte Carlo method as follows. The available measurements for patients and controls were merged and shuffled, and then separated into two groups, having the same number in each group as the original number of patients and controls. The ROC curve and AUC were then recalculated. This procedure was repeated 10,000 times, generating an estimated distribution of AUC under the null hypothesis that patient and control measurements were drawn from the same underlying distribution. An approximate *P*-value for the AUC of the original data was then calculated by determining what fraction of the AUCs found from the shuffled data were larger.

The percentage change of R2 area, latency and duration were compared using unpaired *t*-tests. For all tests a *p* < 0.05 was considered significant leading to rejection of the null hypothesis. Homogeneity of group variances was assessed by Levene's test for equality of variance prior to testing, and *t*-tests which assumed equal or unequal variance of groups selected as appropriate.

Task performance during the pinch grip task was quantified by two measures. *Bias* measured the average difference between force exerted and the target force. *Random error* measured the standard deviation of the difference between the moment-by-moment force exerted and the mean for that target level. In both cases, these measurements were taken over the period 0.6–3.06 s after the cue onset, which was the region used for IMC analysis. Both errors were expressed as a percentage of MVC for that subject.

Signal analysis was performed using custom scripts written in the MATLAB environment (Mathworks Inc., Natick, MA, USA). Statistical analysis used the SPSS 20 statistical package (IBM SPSS, Chicago, IL, USA).

## Results

IMC was investigated in 10 healthy participants and 22 WC patients (*n* = 20 for IMC). The WC patients had scores on the writer's cramp rating scale (WCRS) (27) ranging from 2 to 15 (mean 5.76, SD 3.17).

### Intermuscular coherence (IMC)

Healthy controls and WC patients performed the pinch grip task successfully. Both groups accurately produced the target force at the lowest force level (10% MVC; bias 2.9 ± 1.5% and random error 2.9 ± 1.5%, both given as mean ± SD across the whole population tested). Performance deteriorated at the highest force level (80% MVC), with substantial undershoot of the target and more variable performance (bias −11.5 ± 8.1%, random error 13.1 ± 7.4%). An ANOVA showed a significant effect of force level on both random error (*F* = 29.7, *P* < 0.001) and bias (*F* = 50.9, *P* < 0.001) but no significant effect of group (controls vs. WC patients) or their interaction (*P* >0.1).

**Figure 1:**
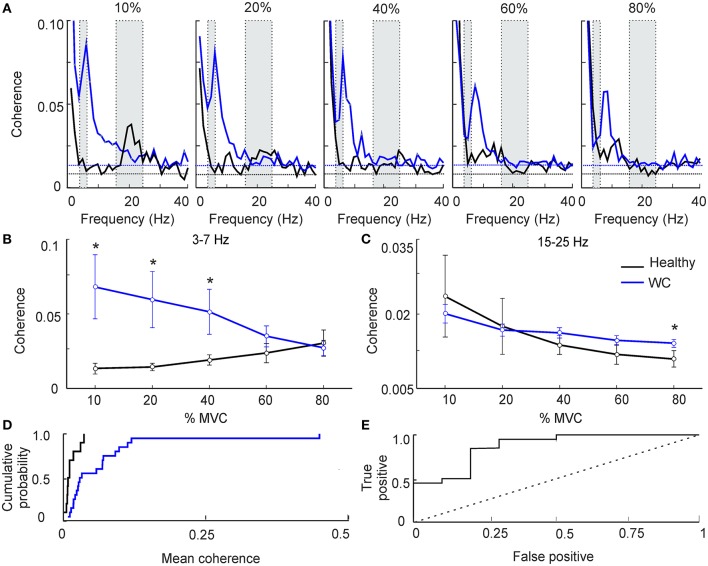
The effect of force level on intermuscular coherence (IMC). (**A)** Intermuscular coherence in healthy participants (black) and patients with writer's cramp (blue) at a range of force levels expressed as a percentage relative to maximum voluntary contraction (MVC). In each plot coherence is averaged across all muscle pairs and across all subjects. The gray rectangular boxes highlight the frequency bands of interest, namely 3–7 Hz and 15–25 Hz. Horizontal dashed lines are the *P* < 0.05 significance levels for WC patients and controls respectively. Note that peak IMC for WC patients, 3–7 Hz at 10–20% MVC, was larger than in healthy volunteers. **(B,C)** Graphs plotting IMC averaged across a frequency band for WC patients (blue) and healthy participants (black) as a function of force level, at 3–7 Hz **(B)** and 15–25 Hz **(C)**. Error bars depict standard error of mean. Stars indicate significant difference between groups at the specified force level (unpaired *t*-test; *p* < 0.05). Note that at low force levels 3–7 Hz IMC was significantly greater in WC compared to healthy participants. **(D)** Cumulative probability distributions of 3–7 Hz IMC in WC patients (blue) and healthy controls (black) during a 10% MVC. **(E)** ROC plot of 3–7 Hz IMC at 10% MVC derived from the cumulative probability distributions in **(D)**, confirming that 3–7 Hz IMC can separate healthy controls from WC patients [area under the curve (AUC) 0.87; *p* = 0.001, Monte Carlo test]. Dotted diagonal line indicates expected result if IMC did not discriminate the groups.

Figure 1 presents results from IMC at various force levels (10–80%), averaged across subjects. As expected in healthy volunteers, at low force levels we observed greater beta band (15–25 Hz) IMC, which declined as contraction strength increased (Figure 1A, black line) (28). WC patients had significant coherence in the beta band, but also had a significant peak in the 3–7 Hz (theta) band that was lower in controls (Figure 1A, blue line). This peak shifted progressively in its frequency as contraction strength increased, until by 80% MVC the peak had almost entirely left the theta band and now overlapped the peak at physiological tremor frequencies (8–12 Hz) seen in healthy subjects. Results are presented averaged across the theta and beta bands in Figures 1B–C. For the theta band, ANOVA indicated a significant effect of subject category (patients vs. controls, *F* = 8.4, *P* < 0.005) but not force level or their interaction (*P* > 0.1). Levene's test revealed homogeneous variances of theta band IMC at all force levels (*p* >0.05) and homogenous variances of beta band IMC at higher force levels (40–80%). WC patients had significantly greater IMC than controls in the theta band at the three lowest forces (Figure 1B, 10–40% MVC; *t*-test, *P* < 0.05). For the beta band (Figure 1C), ANOVA showed no significant effect of subject category (*P* > 0.1); there was an effect of force level (*F* = 3.4, *P* < 0.05) but not the interaction. A dependence of beta band oscillations on force level has previously been reported (29).

Figures 1A,B reveals clear differences in average theta-band IMC between groups; however, this does not provide a measure of how consistent such differences were at the single subject level. To investigate this, we plotted the cumulative probability distributions of IMC averaged in the 3–7 Hz band for WC patients and controls (Figure 1D). These curves were well separated: half the WC patients had IMC values greater than the highest IMC value found in healthy participants (0.03).

To quantify the degree of overlap of these distributions, we then computed the ROC curve (Figure 1E). This was constructed by placing an arbitrary threshold on the IMC, and classifying each subject as “WC patient” if their IMC was above, or “healthy control” if the IMC was below the threshold. For a given choice of threshold, a certain fraction of patients will be correctly classified as patients (the “true positive” rate), and a certain fraction of healthy controls will be misclassified as patients (the “false positive” rate). We measured these classification rates for a range of thresholds. At a very low threshold, all subjects were classified as patients, yielding a 100% true positive rate but also a 100% false positive. For a very high value of threshold, all subjects were classified as healthy controls, yielding 0% for both true positive and false positive rates. The ROC curve plots the changes in true and false positive rates for intermediate threshold values. The area under this curve measures how well 3–7 Hz IMC discriminates the two groups; it was significantly above the chance level of 0.5 (AUC 0.87; *p* = 0.001, Monte Carlo test).

As another method to investigate inter-subject variation, we calculated the correlation between IMC in the theta band and the patient's clinical score on the WCRS. There was no significant correlation (r^2^ from Pearson correlation 0.013 and 0.006 for 10 and 20% force levels respectively, both *P* > 0.05).

**Figure 2:**
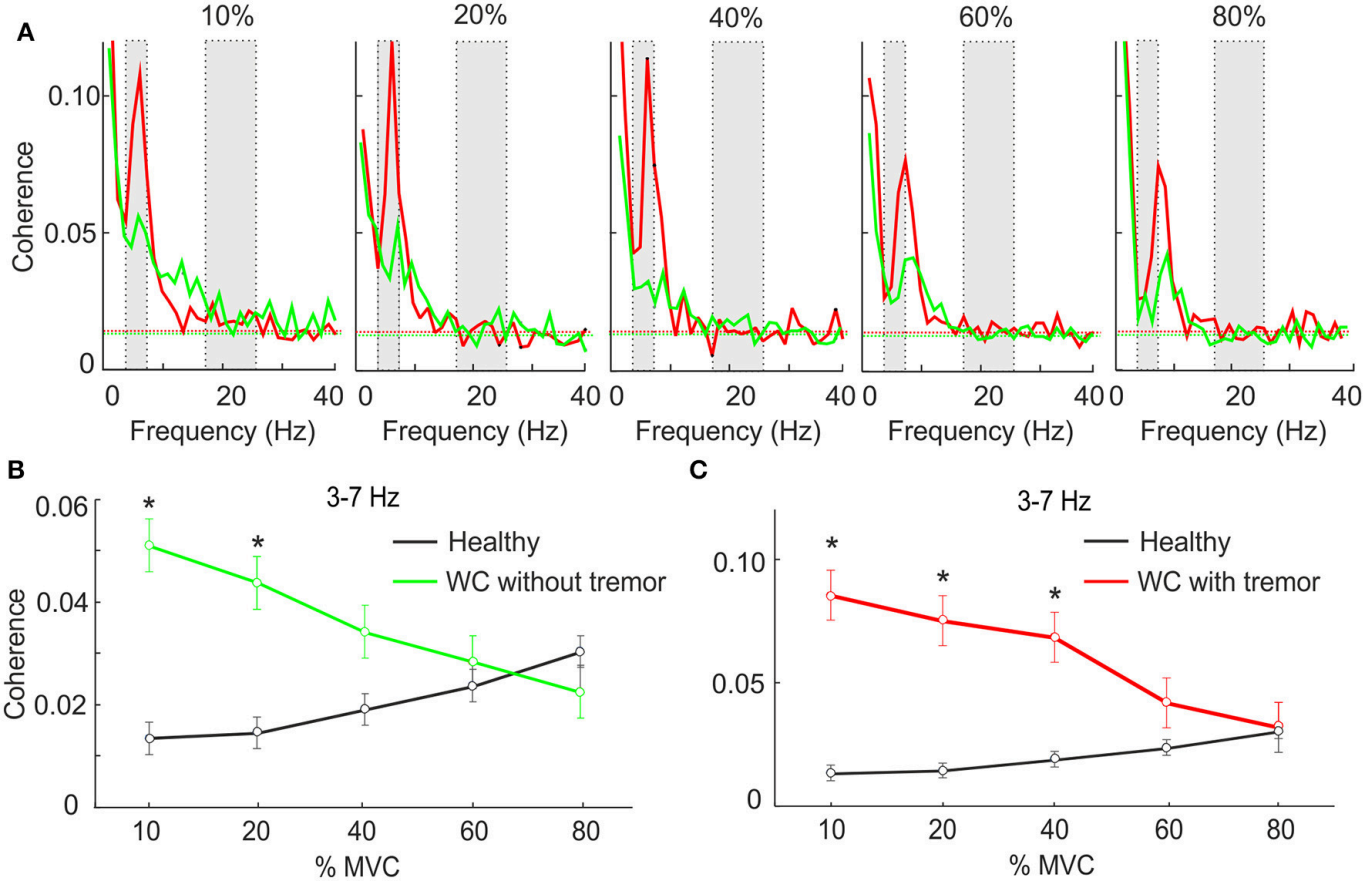
The effect of force level on IMC in WC patients with and without tremor. **(A)** Intermuscular coherence spectra from WC patients with tremor (red) and WC patients without tremor (green) plotted at a range of force levels expressed as a percentage of maximum voluntary contraction (MVC). In each plot coherence is averaged across all muscle pairs and across all subjects. The gray rectangular boxes highlight the frequency bands of interest, namely 3–7 Hz and 15–25 Hz. Horizontal dashed lines are the *P* < 0.05 significance levels. **(B,C)** Graphs plotting IMC averaged across the 3–7 Hz frequency band for WC patients without **(B)** and with tremor **(C)** as a function of force level. Black lines indicate results for healthy controls, for comparison. Stars indicates significant difference between WC subgroup and healthy controls at the specified force level (unpaired *t*-test; *p* < 0.05).

Many WC patients exhibit overt tremor, and we were interested to determine if the theta IMC which we observed was related to this tremor. Accordingly, we separated WC patients according to the presence (*n* = 10) or absence (*n* = 10) of tremor, and averaged IMC at various force levels separately between these groups (Figure 2A). At low forces theta IMC was observed in both groups, although it was larger for those with overt tremor. This finding was replicated when we examined the mean theta band IMC (Figures 2B,C). An ANOVA found that there was a significant effect of group (healthy control, WC with tremor and WC without tremor; *F* = 6.6, *P* < 0.005) but not of force level or the interaction. Pairwise testing indicated a significant difference between both WC groups and healthy controls at low forces (stars in Figures 2B,C; *t*-test, *P* < 0.05). However, there was no significant difference between the WC patients with or without tremor at any force levels (*t*-test, *P* > 0.05).

We also measured the mean power in the 3–7 Hz band in the EMG, as a surrogate marker for tremor amplitude, and calculated the correlation with IMC across all WC patients. There was no significant correlation, whether we used power in individual muscles, or summed across all three muscles available (*r*^2^ ranged from 0.02 to 0.08, *P* > 0.05, Pearson correlation).

### Pre-pulse inhibition of the blink reflex

**Figure 3:**
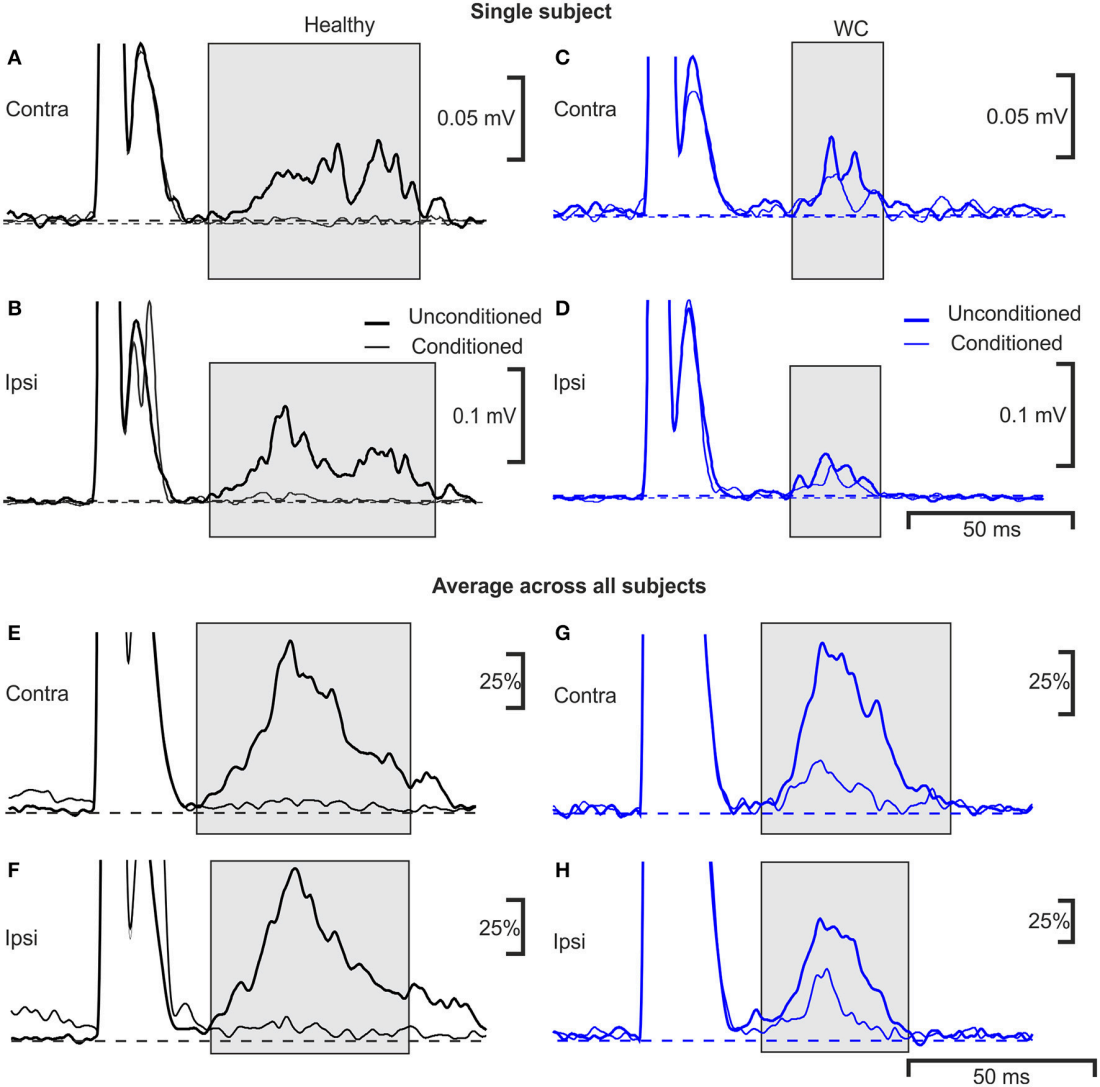
Averaged EMG responses showing pre-pulse inhibition of the blink reflex in a single subject and average across all subjects. Examples of average rectified orbicularis oculi EMG responses from a healthy control **(A,B)** and a patient with WC **(C,D)** recorded during a pre-pulse inhibition testing protocol. Unconditioned responses evoked by stimulating the right supraorbital nerve alone are plotted with a thick line, whereas conditioned blink responses (right median nerve stimulation 100 ms before supra-orbital nerve stimulation) are shown with a thin line. Responses recorded from orbicularis oculi contralateral to the supraorbital nerve stimulus are plotted in **(A,C)** (ipsilateral recordings are plotted in **B,D**). The thin and thick dotted lines show baseline EMG activity of average conditioned and unconditioned blink reflex respectively. **(E–H)** similar traces as **(A–D)**, but now averaged over all subjects recorded (*n* = 10 WC patients, *n* = 9 healthy controls). Individual averages were first normalized by subtraction of the pre-stimulus baseline, and scaling so that the peak in the unconditioned response was fixed at 100%; these normalized responses were then averaged together.

Examples of typical ipsilateral and contralateral conditioned and unconditioned blink responses are illustrated in Figure 3, obtained from an age-matched control (Figures 3A,B) and a WC patient (Figures 3C,D). Traces averaged across all controls, and all WC patients, are shown respectively in Figure 3E–H.

The R2 component of the blink reflex, occurring at latencies of 40–80 ms after the test stimulus to the supra-orbital nerve, was observed in all participants (gray shading). The variances of blink reflex responses were homogeneous (*p* >0.05). However, when ipsilateral blink reflex responses (with and without conditioning) were expressed as ratios their variances were not homogeneous. The mean onset latency of the ipsilateral blink response for WC patients was 42.9 ± 5.7 ms (range 35.9–55.6 ms) and for healthy participants this was significantly shorter at 37.1±3.9 ms (range 29.6–42.5 ms; *p* = 0.020). The duration of the ipsilateral R2 component of the blink response was 44.4±8.3 ms in healthy controls compared to 32.1 ± 6.6 ms in WC patients (*p* = 0.002). Whilst the area under the curve of the ipsilateral R2 response (0.98 ± 1.10 mV.ms; mean ± SD) and contralateral R2 response (0.74 ± 0.68 mV.ms) were both reduced in WC patients compared to healthy controls (ipsilateral 1.6 ± 1.3 mV.ms; contralateral 1.3 ± 1.1 mV.ms), this difference did not reach significance (ipsilateral *p* = 0.33; contralateral *p* = 0.23; unpaired *t-*tests). Similarly, although the threshold for blink reflex to supraorbital nerve stimulation for WC patients (21.5 ± 5.6 mA) was slightly higher than for healthy participants (16.4 ± 4.8 mA), this difference was not significant (*p* = 0.076; unpaired *t*-test). All healthy participants showed a third peak, R3, which was consistently absent in WC patients.

**Figure 4:**
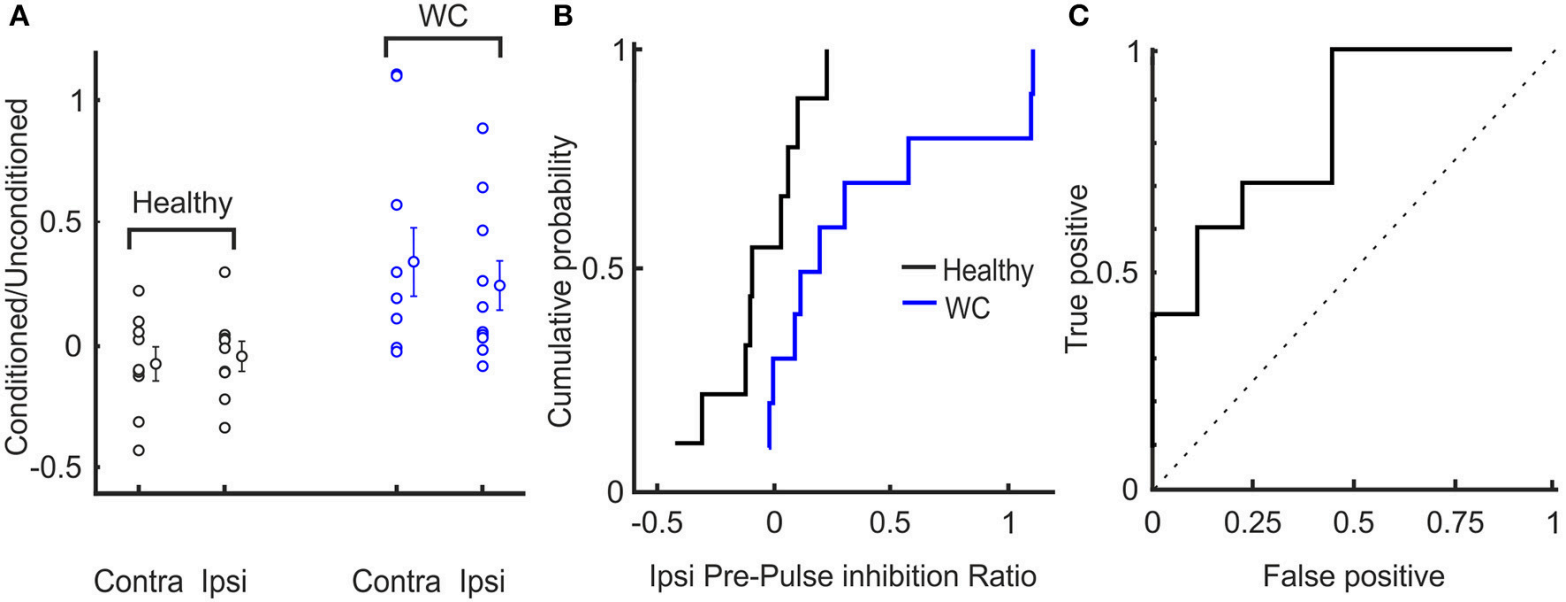
Interindividual variation of pre-pulse inhibition of blink reflex in healthy and WC patients**. (A)** Scatter plot showing distribution of extent of pre-pulse inhibition (ratio of area under the curve above baseline of conditioned to unconditioned reflex) across subjects. The mean and SEM of pre-pulse inhibition of blink reflex between healthy and WC patients are also presented for ipsilateral (ipsi) and contralateral (contra) eyes relative to the side stimulated with the supra-orbital stimulating electrode. **(B)** Cumulative probability plots of the ratio (conditioned/ unconditioned) of pre-pulse inhibition of ipsilateral blink reflex across subjects. **(C)** ROC plot of ratio of pre-pulse inhibition of ipsilateral blink reflex derived from the cumulative probability distributions in **(B)**, confirming that pre-pulse inhibition of blink reflex can separate healthy controls from WC patients (AUC 0.711; *p* = 0.017, Monte Carlo test). Dotted line indicates expected result if no separation of groups was observed.

The extent of inhibition of the R2 response was significantly less for WC than healthy controls (conditioned/unconditioned R2 area ratio for ipsilateral reflex: 0.34 ± 0.43 vs. −0.07 ± 0.20, *p* = 0.019; for contralateral reflex: 0.24 ± 0.31 vs. −0.04 ± 0.17, *p* = 0.029; Figure 4A). The cumulative probability curves for ipsilateral PPI of blink reflex in healthy and WC patients (Figure 4B) showed a clear difference and the corresponding ROC curve revealed an AUC value of 0.711 (*p* = 0.017, Monte Carlo test) (Figure 4C). There was no correlation between the clinical score on the WCRS and the ipsilateral R2 area ratio (*r*^2^ = 0.363, *P* > 0.05).

## Discussion

Here we present evidence that brainstem pathways may be involved in the pathophysiology of WC, based on results from two very different electrophysiological measurements in a cohort of patients with WC (*n* = 20 for IMC experiments and *n* = 10 for blink reflex experiments). Although the numbers of patients available for study was relatively small, effects were robust and statistically significant, providing confidence that our results are likely to reflect a genuine feature of this disease. We did not match the control groups for age and gender in our study, which could be considered a limitation. However, we have previously shown that inter-muscular coherence does not vary with age or gender (19).

The blink reflex is a physiological protective response, which is dysfunctional in various movement disorders (11–13). Inhibition of the R2 component of the blink reflex is mediated largely via the PPN and when tested using a PPI protocol is reportedly diminished in upper and lower limb dystonia (10). PPI of the blink reflex is therefore a marker of sensory-motor gating at the level of the brainstem (30). Whilst inhibition of the R2 response may be reduced in some focal dystonias [e.g., blepharospasm and cervical dystonia (11–13)], the only previous study in WC concluded that PPI was normal (11). In contrast to that small study in three patients, we showed reduced PPI in our larger cohort using similar methods. However, the variability in PPI among our sample of WC patients is perhaps too great for it to be used as a diagnostic test (AUC of ROC 0.65).

Our observation that the R2 component of the blink response is not attenuated at the inter-stimulus interval tested might suggest a failure of inhibition and thus hyperexcitability within the brainstem network subserving PPI. Surprisingly, hyperexcitable blink responses were not observed. Instead stimulation thresholds for eliciting blink responses were slightly increased, and R2 responses were slightly reduced in WC patients, although neither difference reached significance. The results of our PPI experiments could thus be ascribed to a floor effect whereby unconditioned R2 responses were already strongly inhibited, and thus additional inhibitory input could have no further effect. GABAergic inhibitory interneurons play a pivotal role in synchronizing oscillatory activity within neuronal networks (31, 20). If there is indeed increased inhibition in the brainstem in WC, this may also increase oscillatory activity (32–34), potentially explaining the presence of increased theta frequency IMC in WC patients. The *globus pallidus interna* is one potential source of increased inhibitory input to the brainstem blink reflex network (35). Pathologically increased connections between pallidum and brainstem nuclei (including PPN) have been described in cranio-facial dystonia (36).

Several previous studies have investigated IMC in patients with WC. One report in eight patients showed clear coherence at both beta (~20 Hz) and gamma (~40 Hz) bands, which was similar to controls (37). Another study in five patients showed significant IMC at 11–12 Hz only in WC patients with tremor (38). In patients with DYT 1 gene mutations and limb dystonia (*n* = 12), two of which had WC, significant 3–7 Hz IMC was present in 80% of patients, although the IMC difference compared with controls did not reach statistical significance (16). By contrast, in our work 3–7 Hz IMC was substantially larger in WC patients than controls. Several potential methodological factors may explain this contradictory picture. We used a tonic contraction task, and focused our analysis on the stable hold period; inclusion of phasic periods of contraction or relaxation can result in low frequency coherence as an artifact of covarying activity during task performance (22). The force of muscle contraction and location of recording electrodes can also contribute to variation in IMC, as observed when assessing inter-individual and inter-session differences in IMC (19). We used a systematic approach to controlling the force of contraction, testing a range of force levels in a large cohort of patients. Our findings are therefore likely to be more robust compared with those reported previously.

Importantly, we found that enhanced theta band IMC was a consistent finding in WC patients, even in those without overt tremor. This suggests that patients with or without tremor show similar pathophysiology, but are simply at different ends of a disease spectrum. Whether centrally-generated oscillations in muscle activity produce mechanical tremor depends on multiple factors, such as the low-pass filtering effects of muscle twitch tensions and limb inertia (39). Whilst enhanced tremor may be an important factor contributing to the disability experienced by the patient, it does not seem to indicate a key difference in central processes.

### Origins of theta-band oscillations/intermuscular coherence (IMC)

Recordings from deep brain stimulating electrodes in patients with Parkinson's disease reveal that the PPN can generate local field potential oscillations in the theta frequency range (32–34). Similar PPN oscillations can also be generated *in vitro* from rat brainstem slices following the application of cholinergic agonists (40). These observations are of particular interest given our finding of abnormal blink reflex PPI, which is thought to depend on the PPN. However, the mere existence of theta oscillations from a particular nucleus in Parkinson's disease does not necessarily ensure a common origin of similar oscillations in WC. For example, other sub-cortical structures including the red nucleus (41), nucleus incertus (42), ventral tegmental area (43), and cerebellar deep nuclei (44) have all been reported to generate theta oscillations, although this is usually considered in the context of synchronization with the widely reported theta activity in the hippocampus (45). Nevertheless, if the PPN (or any other subcortical structure) is the origin of the enhanced theta band IMC which we observed, then we must explain how these oscillations propagate to hand and forearm muscles. One possibility is via connections to primary motor cortex and thence the corticospinal tract. Consistent with this possibility, a previous study reported significant CMC in the theta band between contralateral sensorimotor cortex and forearm EMG in WC patients (17). That study made recordings while WC patients wrote, and the coherence at 3–7 Hz was assumed to represent the frequency of the writing movements. By contrast, our recordings were made during a tonic grasping task, which had no requirement for rhythmic motor outflow, suggesting that the oscillations which we observed were the consequence of a specific central oscillator. Importantly, the existence of CMC does not necessarily demonstrate a cortical source for an oscillation. For beta oscillations, we have previously demonstrated that sensory as well as motor pathways may contribute to CMC (46–48). Even a sub-cortical rhythmic drive to muscle, unrelated to cortical activity, could generate oscillatory sensory feedback which might manifest as significant CMC.

At the present time, we therefore cannot rule out a corticospinal source of the theta oscillations observed in WC, but a sub-cortical source, such as the PPN remains a possibility. We have previously shown that the primate reticular formation makes reticulospinal connections to spinal motoneurons and interneurons involved in hand function (49, 50). Given the PPN provides extensive input to the reticular formation (51, 52), reticulospinal propagation of these oscillations would therefore be consistent with known circuits.

### Utility of IMC as a potential diagnostic marker

As well as providing insight into underlying mechanisms, our study suggests that IMC might be further developed as a useful diagnostic tool in focal task-specific dystonia. Theta band IMC was significantly higher in WC patients compared to healthy participants, but this was not just a statistical effect observable at the population level. Use of theta band IMC could give the clinician greater confidence when confronted with a potential functional/psychogenic focal movement disorder mimicking WC. Further prospective studies of patients on first referral of task specific focal dystonia and other tremor/movement disorders are warranted to test further the utility of this approach.

## Conclusion

In this study, we provide evidence that both PPI of the blink reflex and theta band IMC are disordered in WC patients. A deficit in PPI definitely implicates a brainstem abnormality, and it is possible that brainstem circuits could also be involved in the circuit generating a high theta IMC. There may therefore be a role for the brainstem and its connections in the pathogenesis of WC. Finally, our research suggests that the theta-frequency IMC is a reliable electrophysiological biomarker for WC.

## Author contributions

SC: study concept and design, acquisition of data, analysis and interpretation, critical revision of the manuscript for important intellectual content. RS, PC, and MRB: acquisition of data, analysis and interpretation, critical revision of the manuscript for important intellectual content. ST and SS: patient diagnosis, critical revision of the manuscript for important intellectual content. HK and SNB: study concept and design, acquisition of data, analysis and interpretation, critical revision of the manuscript for important intellectual content, study supervision.

### Conflict of interest statement

ST: Advisory board member of Sun Pharma. HK: Advisory board member of Allergan India. The remaining authors declare that the research was conducted in the absence of any commercial or financial relationships that could be construed as a potential conflict of interest.
